# Adherence to prophylaxis in adolescents and young adults with severe haemophilia A, a qualitative study with patients

**DOI:** 10.1080/21642850.2018.1493384

**Published:** 2018-09-24

**Authors:** S. van Os, N. Troop, N. Ryder, D. P. Hart

**Affiliations:** a Department of Applied Health Research, University College London, London, UK; b Psychology and Sport Sciences Department, School of Life and Medical Sciences, University of Hertfordshire, Hertfordshire, UK; c The Royal London Hospital Haemophilia Centre, Barts Health NHS Trust, London, UK; d Barts and The London School of Medicine and Dentistry, QMUL, London, UK

**Keywords:** Adherence, haemophilia, prophylaxis, self-management, adolescents, young adults, personalised treatment

## Abstract

**Introduction:** Reported levels of adherence to prophylaxis among young people with haemophilia (YPH) vary widely and are predominately based on estimations made by healthcare professionals and parents. Reasons for (non)adherence among YPH in particular have not been evidenced.

**Aim:** to examine experiences in relation to prophylaxis with YPH themselves, and barriers and facilitators to their adherence.

**Methods:** 11 Participants were recruited in five haemophilia centres across England and Wales. All patients who met the inclusion criteria (aged 12-25, diagnosed with haemophilia, on prophylaxis) were approached during a routine check-up appointment, and all participants who agreed to take part were interviewed. Interviews were audio recorded, transcribed and analysed using Interpretative Phenomenological Analysis.

**Results:** Self-reported adherence to prophylaxis was good. Few participants admitted to intentionally skipping injections although they reported sometimes forgetting. However, due to the increasingly personalised and flexible approach to prophylaxis, adherence is not straightforward to define. Barriers to adherence included a busy lifestyle, dislike of the intravenous injection, venous access issues, anxiety or stress and being out of one’s normal routine. Support was an important facilitator to adherence, including support from health professionals at the haemophilia centre as well as friends. Parents appear to be very involved with their child’s haemophilia management, even after they leave home.

**Conclusion:** What this study adds is that the increasingly flexible and personalised approach to managing prophylaxis in haemophilia may sometimes lead to confusion around treatment frequency and dosing. This may lead to accidental non-adherence, which is distinct from both skipping and forgetting. Advice from haemophilia teams may not always be consistent and is likely to be interpreted differently by different individuals. Some additional training and education of patients and their families to increase their knowledge and skills around prophylaxis may reduce this confusion and therefore is likely to improve adherence further.

## Introduction

Haemophilia is an inherited bleeding disorder that occurs mostly in males and is caused by a deficiency in one of the coagulation (blood clotting) factors. According to the United Kingdom Haemophilia Centre Doctors’ Organisation *(*UKHCDO) Annual Report, 2015/2016 there are approximately 7,700 people in the UK with Haemophilia A (deficiency in factor VIII) and 1,707 with Haemophilia B (deficiency in factor IX). Haemophilia is classified as mild, moderate or severe based on the concentration of factor measured via a blood test (Bolton-Maggs & Pasi, 2003). This study is concerned with patients with severe haemophilia (approximately 1/3 of those with haemophilia) where the concentration of factor in the blood is generally less than 1%. This results in a greater risk of spontaneous bleeding and excessive bleeding after injuries, accidents and surgery.

Haemophilia is treated by replacing the deficient coagulation factor through intravenous injections of factor concentrate. Treatment can be on-demand, where medication is used to treat bleeding; or preventative, where treatment is used to increase the concentration of factor in the blood to reduce risk of bleeding (Collins, 2012). Most patients with severe haemophilia in the UK follow a preventative treatment regimen (known as prophylaxis). For each patient on prophylaxis lifetime healthcare costs are estimated at £5.98 million for haemophilia A, and £2.47 million for haemophilia B (Miners, 2009).

Prophylaxis reduces joint bleeds and resulting joint damage (Fischer et al., 2002; Manco-Johnson et al., 2007) as well as intracranial bleeds (Ljung, 2009; Witmer et al., 2011), whilst improving quality of life (Richards et al., 2010). Initiating prophylaxis at an early age, often shortly after diagnosis, is the optimal form of treatment for severe haemophilia (Coppola, Di Capua, & De Simone, 2008). Parents are trained to give the injections to their child at home, while children are encouraged to help from a young age (e.g. mix up treatment ready for the injection, clean the skin, etc.), and gradually take over the responsibility for their treatment. Haemophilia teams generally aim for patients to do their own injections by the time they start senior school. However, the age at which patients become responsible varies widely (Khair, 2013).

In previous decades haemophilia A prophylaxis regimens were based on patients’ weight and aimed to reduce the risk of bleeding by increasing the trough level above 1 IU dL ^-1^, thus converting the bleeding phenotype from severe to moderate (Ahlberg, 1965; Nilsson, Berntop, Lofqvist, & Pettersson, 1992). Although this strategy was proven to be highly successful in long-term follow-up studies (Fischer et al., 2002; Nilsson, 1993), it is recognised that some patients bleed despite having a trough level above 1 IU dL ^-1^ (concentration of factor in the blood of 1% of normal), whereas others do not bleed even with an undetectable trough level (Collins, 2012). It is likely that the factor level required to prevent bleeding varies between patients, and that 1 IU dL ^-1^ is therefore not necessarily an appropriate target for all patients (Den Uijl et al., 2011). In recent years regimens have become more flexible, and many patients follow a more individualised treatment regimen, designed around their lifestyle, planned activities, individual bleeding pattern, condition of their musculoskeletal system, and measurement of coagulation factor in their blood (Collins, 2012; Fischer, 2012; Makris, 2012). In addition, many patients in the UK are encouraged to top up with an additional injection in advance of physical activity if they feel their usual regimen will not afford them enough protection.

Patients’ adherence, the extent to which they follow their agreed treatment regimen, has considerable influence on treatment efficacy. Non-adherence (i.e. not keeping to the agreed frequency and/or dosing and/or timing of prophylactic injections) increases the risk of spontaneous bleeds (Collins et al., 2009; Hacker, Geraghty, & Manco-Johnson, 2001), which increases treatment costs (Panicker, Warrier, Thomas, & Lusher, 2003), and may result in joint damage leading to poorer physical and emotional wellbeing (du Treil, Rice, & Leissinger, 2007; Marilyn J Manco-Johnson et al., 2007). In addition to the immediate costs associated with treating bleeds (on-demand treatment, potential hospitalisation, clinic visits, etc.), they may also lead to increased future care-costs due to disability. Even low levels of non-adherence can result in significant medical problems and permanent disability (du Treil et al., 2007).

A recent review (Schrijvers, Uitslager, Schuurmans, & Fischer, 2013) identified that there is little research on adherence among people with haemophilia, with most studies being of poor quality (based on sample size, methodology, and bias). Nevertheless, in their review (Schrijvers et al., 2013) suggest that the key motivators of adherence to prophylaxis are experience of symptoms, a positive belief of necessity of prophylaxis and a good relationship with the healthcare provider. They suggest that the most important barriers to adherence are infrequent or absence of symptoms and increasing age.

A recent study with a large sample of young people on prophylaxis (representing nearly 20% of the whole target population) who were recruited from 13 haemophilia centres across England and Wales (van Os, Troop, Sullivan, & Hart, 2017) found adherence to be good (over 80%). In this study adherence was predicted by a greater necessity concern differential (where patients’ perceived the need for prophylaxis to be greater than their concerns over taking it), and a positive expectancy of the effectiveness of prophylaxis, good social support and a stronger (negative) emotional reaction to having haemophilia were also associated with better adherence. However, it is important to note that measures that assess adherence in haemophilia (including the widely used VERITAS-Pro: Duncan, Kronenberger, Roberson, & Shapiro, 2010), are based on a traditional approach to treatment. This means their utility in the context of an increasingly flexible, personalised treatment is somewhat limited. For instance, patients who have agreed a more flexible approach with their haemophilia doctor, and are therefore not required to contact the haemophilia centre before making one-off adjustments to their regimen, may be considered non-adherent by the VERITAS-Pro. Furthermore, the range of predictors of adherence used in van Os et al.’s (2017) study (including beliefs about medicines, social support and outcome expectancies) was limited to those previously identified as predictors of (non-)adherence in haemophilia and other chronic conditions (Gallant, 2003; Horne & Weinman, 1999; Iannotti et al., 2006; Llewellyn, Miners, Lee, Harrington, & Weinman, 2003). Therefore, it may not have included all relevant predictors of adherence to prophylaxis among young people with haemophilia. This is particularly important given the (apparently) high rate of adherence to prophylaxis in haemophilia, which is much higher than levels of adherence reported in other chronic conditions.

Therefore, a more exploratory study is required to examine what makes young people with haemophilia, and the way in which they adhere to their treatment, different from young people with other chronic health conditions. Previous adherence research has highlighted that patient perceptions and experiences in relation to their condition, treatment and support strongly influence their adherence (DiMatteo, 2004; Horne & Weinman, 1999; Iannotti et al., 2006; Griva, Myers, & Newman, 2000; Ott, Greening, Palardy, Holderby, & DeBell, 2000). Therefore the present study examines perceptions and lived experiences in relation to haemophilia and prophylaxis, in order to better understand of what drives (non-)adherence among young people with severe haemophilia in the UK. The chosen qualitative approach for this study, Interpretative Phenomenological Analysis (IPA), combines psychological, interpretative and idiographic components. It aims to offer insights into how a given person, in a given context, makes sense of a given phenomenon (Smith, 2010). Therefore its focus is on ‘*exploring experience in its own terms’*, without reducing it to ‘*predefined or overly abstract categories’* (Smith, 2010). This approach will allow an in-depth investigation of adherence, without forcing participants merely to reinforce pre-defined ideas and expectations. IPA employs a ‘double hermeneutic’ in which the researcher tries to make sense of the participant trying to make sense of their experiences (Smith, 2003; Smith, Flowers, & Larkin, 2009). Therefore the study does not require participants to already have clearly defined ideas about their adherence. In order to make sense of the participant’s personal world the researcher engages in a process of interpretative activity in which both the participant’s and the researcher’s own conceptions play an important role.

## Methods

### Recruitment

IPA studies usually employ a fairly homogenous sample, drawing on the accounts of a small number of people who have certain experiences in common (Reid, Flowers, & Larkin, 2005). Therefore inclusion criteria for participation in this study were very specific; participants had to be aged 12–25 years old, have been diagnosed with severe haemophilia, and following a prophylactic treatment regimen. Participants were recruited in five haemophilia centres across England and Wales. Initial screening was carried out by haemophilia doctors and nurses, to identify all participants who met the inclusion criteria. All eligible patients were approached by a research nurse while they attended routine check-up appointments. They were given an information sheet about the study to take away, and encouraged to discuss the study with others. All potential participants (or parents of those aged 17 or younger) who had taken the information sheet home were then contacted by a research nurse to check whether they would like to take part. All participants who agreed to take part were then contacted by the researcher to arrange a date and location for the interview. Informed written consent was obtained on the day of the interview from all participants, and in addition from parents of participants aged 17 or younger.

### Participants

Participants were 11 males with a mean age of 18.8 years (SD = 5.0), see Table 1 for details.

**Table 1.** Young people with haemophilia A interview study participant characteristics.

Participant code	Ethnicity	Age	Treatment regimen
1	White British	17	Three times per week
2	White British	24	Activity based
3	White British	21	Twice per week Long-acting
4	White British	12	Three times per week
5	White British	25	Three times per week
6	Asian Pakistani	22	Alternate days
7	White other European	13	Twice per week Long-acting
8	White British	12	Alternate days
9	White British	16	Daily
10	White British	21	Three times per week
11	White British	24	Three times per week

### Interpretative phenomenological analysis (IPA)

IPA is a qualitative research approach that aims to offer insights into how a given person, in a given context, makes sense of a given phenomenon (Smith, 2010). It is concerned with personal perceptions or accounts of experiences, rather than an attempt to produce objective statements of experiences. The IPA research exercise is a dynamic interpretative process, where both the participant’s and researcher’s perceptions play an important role. In this process different interpretative stances are possible, combining empathic hermeneutics (trying to understand what it is like from the participant’s point of view) with questioning hermeneutics (asking critical questions from the text to interpret people’s mental and emotional state from what they say).

Yardley’s (2000, 2008) ‘*Characteristics of good qualitative research*”’ were used as the guiding principles to ensure this study meets standards in relation to rigour, validity and credibility. Key considerations in the research process were the transparency of data presentation, reflexivity around the researchers own assumptions, and consideration of alternative perspectives. After examining themes, and convergence and divergence of themes, and after immersion in the data, those themes that emerged most strongly from the interview data and that were relevant to the research questions were reported.

### Data collection

IPA studies aim to analyse in detail how individuals perceive and make sense of experiences, and therefore require a flexible approach to data collection. Semi-structured interviews are considered a good data collection method for IPA studies, as they facilitate a more informal and free-flow interview, which enables the researcher to follow cues from participants and probe areas of interest that appear particularly relevant to each participant’s experiences.

A semi-structured discussion guide was developed based on literature in relation to treatment adherence among young people diagnosed with chronic health conditions (Alvin, Rey, & Frappier, 1995; Arias Llorente, Bousoño García, & Díaz Martín, 2008; Iannotti et al., 2006; La Greca & Bearman, 2002; La Greca et al., 1995; Salema, Elliott, & Glazebrook, 2011; Shaw, 2001), including haemophilia (De Moerloose, Urbancik, Van Den Berg, & Richards, 2008; du Treil et al., 2007; Duncan et al., 2010; Khair, 2013; Llewellyn et al., 2003; Thornburg, 2008), and guidance from Smith and Osborn (2003). The draft discussion guide was then finalised using feedback collected from focus groups with patients and haemophilia healthcare professionals (HP). Questions were delivered in an open-ended and non-directive style to encourage participants to share their story in their own words. Participants were reassured that information given would be confidential and not shared with anyone involved with their care.

To build rapport each interview started with some questions about what it is like to live with haemophilia. To gain insight into their experiences and perceptions in relation to prophylaxis, participants were invited to describe their treatment regimen, their feelings about their treatment, potential barriers and facilitators to their adherence, and social support they receive in relation to their haemophilia and treatment. The order in which the above subjects were discussed was flexible, and driven by participants themselves. Interviews lasted between 35 and 95 minutes and were audio recorded, transcribed verbatim, and analysed following the Interpretative Phenomenological Analysis (IPA) principles and guidelines (Smith, 2003; Smith & Osborn, 2003).

### Data analysis

IPA emphasises that the process of discovering themes is based on the researcher being engaged in a double hermeneutic (Smith et al., 2009), with the aim of making sense of the participant attempting to make sense of their experiences while recognising that individuals construct meanings within both a social and personal world.

The transcription of the interview recordings, seen as the first phase of analysis in IPA, consisted of three stages: firstly an ‘everything audible’ version was produced to ensure that future transcriptions stemmed from the maximum possible transcribed content. In the second ‘cleaned and confidentialised’ transcription irrelevant noises and identifying information were removed. As none of the participants took the opportunity to review their interview transcript, validation was carried out by an independent researcher who read the transcripts while listening to the recordings. The main researcher then re-read the transcripts to ensure that all identifying information was removed, and the transcripts were ready for analysis.

The analysis process started by making comments and annotations (in NVivo) while reading and re-reading the transcript. The researcher then coded the transcript while re-reading the transcript along with the comments and annotations. The coded transcript was then reviewed, and the coding was refined where needed. The NVivo coded transcript was then exported, creating an Excel spreadsheet with one line for each code, and columns providing more information about the code (code name, frequency the code was used during coding, example quote, and notes/reflections including bracketing off personal interpretations that may influence the analytical process, as well as themes to which the code may belong. The themes were then reviewed to identify overarching themes, which were reported in an additional column. Throughout this process the researcher continually returned to the transcripts to ensure that the superordinate themes still reflected what participants had actually said. Finally, the overarching themes were refined and presented in a table together with each of their subordinate themes. As recommended by Smith and Osborn (2003), it was decided to firstly conduct IPA on a single interview in its entirety. The remainder of the transcripts were then taken through the analytical process together. To ensure rigour, validity and credibility, an independent researcher, who did not have any prior knowledge of haemophilia or treatment adherence, then reviewed the subordinate and superordinate themes, and the way they related to the initial transcripts and each other. Questions raised during this process highlighted areas that needed specific attention in the write-up of results to ensure clear and transparent reporting of the results.

## Ethics statement

Ethical approval was obtained from the NHS National Research Ethics Service Committee, London – City Road & Hampstead (NRES, Ref: 12/LO/2030).

## Results

### Superordinate and subordinate themes

Participants’ accounts clustered around four superordinate themes, which are shown in Table 2 together with their related subordinate themes. The table also shows for which participants each of the subordinate themes was relevant.

**Table 2.** Young people with haemophilia interview study superordinate and subordinate themes for each participant.

Superordinate	Subordinate themes	Participants										
		1	2	3	4	5	6	7	8	9	10	11
1. Balance between good self-management and living the life you want	1.1 Haemophilia, bleeds and pain are part of life and who I am.	x		x	x	x	x	x	x	x	x	x
	1.2 Avoiding risk is key	x	x	x		x			x		x	x
	1.3 Patient is haemophilia expert	x	x	x	x		x	x	x		x	
	1.4 I tailor my treatment around my lifestyle.	x	x		x	x		x	x	x	x	
2. Perceptions, barriers and facilitators.	2.1 Barriers to adherence.	x	x				x	x			x	x
	2.2 Haemophilia and treatment related anxiety.	x	x	x	x	x	x	x	x	x	x	x
	2.3 Non-adherence is usually due to forgetting	x	x	x	x	x	x	x	x	x	x	x
	2.4 Taking treatment is inconvenient and no fun.		x		x	x	x	x		x	x	x
	2.5 Taking treatment is part of my routine.	x	x	x	x	x	x	x	x	x	x	x
	2.6 Treatment protects me so I can live a normal life.		x	x		x	x			x	x	x
3. Support from family, friends and the haemophilia centre keeps me on track.	3.1 Support from mum and dad is key.	x	x	x	x				x	x	x	
	3.2 Social and peer support.	x		x	x			x	x	x	x	
	3.3 The staff at the haemophilia centre encourage me to keep to my treatment regimen.	x	x	x	x		x	x	x	x	x	

#### Theme 1: Balance between good self-management and living the life you want

This theme encapsulates the impact haemophilia has on day-to-day life. Often diagnosed at a young age, haemophilia is not only a lifelong health condition, it is part of who patients are and the way they live their lives. Participants described how they were often in and out of hospital as children, resulting in school absence, which for some had a negative effect on academic and professional opportunities. They also described how they had missed out on social occasions and opportunities to make friends because they were often excluded from activities that were deemed too risky (such as playing football). The young people involved in this study vocalised a strong desire to live a ‘normal’ life, and rely on their prophylactic treatment to offer them the protection they need to pursue and achieve life goals, and engage in activities they enjoy. In recent years the focus in haemophilia care in the UK has been moving towards a more individualised approach (Richards et al., 2010). This means that in many cases haemophilia teams agree a treatment regimen that is tailored to the individual patient, based not only on clinical considerations such as bleeding phenotype (individual tendency to bleed) and pharmacokinetics (the movement of a drug into, through, and out of the body), but also the patient’s lifestyle and physical activities (Ar Muhlis, Vaide, Berntorp, & Björkman, 2014; Gringeri, Doralt, Valentino, Crea, & Reininger, 2016).

*Subtheme 1.1: Haemophilia, bleeds and pain are part of life and who I am*. As haemophilia is usually diagnosed in the first few years of life, patients have always lived with the condition and it is therefore part of their identity.

I just kind of accept it as a part of who I am and what I’ve got. (P.1, 17 years old)

Most participants appeared to accept haemophilia-related issues (e.g. bleeds, pain, and joint damage) as part of their life. They tend to take a ‘you just have to get on with it’ approach, and do not dwell on these issues.

It’s okay, the only thing I’m really worried about is when I have to inject myself but then I think that everyone with haemophilia has to do it. (P.8, 12 years old)

*Subtheme 1.2: Avoiding risk is key.* It can be challenging to strike a balance between living a normal life while avoiding situations that increase the risk of bleeding. However, it seems that there are many different interpretations of what ‘avoiding risk’ is, and how this influences the way you live your life. Different families and individuals deal with haemophilia in different ways, and perceive the potential risks associated with activities differently. However, managing risk is an important part of life for all young people involved in this study. For some, this means that as long as they have taken treatment, they feel protected while they get on with the life they want to live.

If I am doing anything active obviously I would have always had my medication uhm. I don’t really leave anything to chance. Uhm so as long as I’ve had that then there is not a huge panic uhm but obviously if I have broken a bone then you’re gonna have to go to hospital either way uhm. (P.2, 24 years old)

For others managing risk has a more central role in their lives, where each activity is risk assessed, and where limitations to what they are able to do are an important consideration in everyday life.

But knowing my limitations is definitely, ah, a very important part of having haemophilia, cos if you go past what you’re limited to be able to do, then you are gonna cause more damage. (P.1, 17 years old)

In particular, participants who live an active lifestyle feel that taking treatment in preparation of activities is crucial in reducing the risk of activity-related bleeds. This means that they often tailor their treatment by taking top-ups in addition to their regular prophylaxis, to ensure they are covered while being physically active.

Recently I’ve done a 10k run and I knew that the week before I’m training for it, make sure you’ve given yourself plenty of factor, maybe increase the dosage a little because you’ve got a big run coming ahead and if something were to happen or generally you’re going to ache after the run anyway so it would be good to have that factor in your system and a good healthy amount in that week before to get you prepared. So if I did miss something, which I didn’t, I would have been worried just in case something happens or I get a cut or something or I bang my head or something and you keep on bleeding and bleeding, whereas if you were prepared then you wouldn’t bleed as much. (P.10, 21 years old)

It is interesting that patients’ perceptions of advice from their haemophilia centre differs widely, with some suggesting that they feel that they are strongly discouraged from getting involved with sports, whereas others feel that they are encouraged to be active and fit. This could be because different haemophilia centres give different advice, but could also be due to different interpretations of the same advice. This suggests that healthcare professionals (HP) need to consider carefully how the advice they give is understood and implemented. Particularly as individual circumstances, family dynamics and personalities will influence the way in which patients and their families take on advice.

I think there’s a couple of haemophilia centres who aren’t handing out, just aren’t really handing out treatment. They’re just saying don’t do sport, if you continue to do it we don’t give you the treatment. Which is a shame to hear because I know all the centres <in this area> encourage sport and an active lifestyle. And you know I’ve learned since I was swimming at a high level that’s when all my problems stopped. That coupled with the medication. It’s been super fit, super healthy, treating the haemophilia with a bit of respect. (P.2, 24 years old)

As illustrated above feedback from several participants in this study suggests that being fit, healthy and strong helps to manage haemophilia and reduces bleeding. Many haemophilia centres advise their patients to take up swimming as it is a non-weight bearing activity that can help patients to stay fit and strong without risk of injury. Several participants in this study mentioned that they swim regularly, or are intending to take up swimming in the near future.

If I could play football like you run around for like ninety minutes, that could affect me like the next day because I could have sore ankles, because swimming’s not weight-bearing it’s ideal. (P.11, 24 years old)

*Subtheme 1.3: Patient is haemophilia expert.* In particular, participants aged 18 and older described how they feel that they are experts in haemophilia. They have learned how to recognise the symptoms of a bleed, they know what kind of activities increase the risk of bleeding, how to tailor their treatment in preparation of physical activity, and they know how to treat themselves if a bleed does occur.

I think, I’m also haematologist [laughs] because I had so much experience of these things but obviously I feel I had to teach myself, I’ve improved myself a lot and I’ve gained enough confidence too. (P.11, 24 years old)

As haemophilia is a rare condition, several participants described situations in which (non-haemophilia) HPs did not know how to deal with their condition, or gave them incorrect advice (e.g. some were prescribed nonsteroidal anti-inflammatory drugs, which can worsen bleeding problems). For some participants, these experiences encouraged them to become more self-sufficient*.* Even those participants who did not describe themselves as experts appeared very confident about their abilities to manage their haemophilia, and were knowledgeable about the way factor replacement treatment works.

Cos there’s nothing high-risk about sleeping. The only time I’ll take it in the evening is if I’ve had like 2000 units on a Monday and then I am doing something active on the Tuesday evening then I’ll maybe have a top-up of a 1000 that evening. (P.2, 24 years old)

*Subtheme 1.4: I tailor my treatment around my lifestyle.* In recent years the focus in haemophilia care has been moving towards a more individualised approach, where patients are encouraged to tailor their treatment around their lifestyle. So instead of the standard 3 injections per week (usually on Monday, Wednesday and Friday in the morning), patients may tailor the treatment frequency and dosing around their activities and lifestyle. Many patients also take additional treatments (top-ups) to cover themselves for ad-hoc activities on days that fall outside of their usual prophylactic regimen. The majority of participants in this study tend to tailor their treatment using top-ups. However, two participants described more flexible regimens that they tailor on a daily basis around their very active lifestyles.

I can have some pretty intense weeks. Where I maybe go over, uhm not my quota because there is not really a quota for it, but kind of if I’ve taken it every day and I’ve had couple of days where I’ve gone 1000 and 2000. If I have a day or a few days where I am not really active then I sort of try and knock it back quite a bit. (P.2, 24 years old)

#### Theme 2: Perceptions, barriers and facilitators

This theme encapsulates barriers to adherence as described by the participants in this study, and may therefore not include all possible barriers to adherence to prophylaxis that young people may experience.

*Subtheme 2.1: Barriers to adherence.* Most young people involved in this study have a busy lifestyle, which can make it hard to fit treatment in. Many leave home early in the morning to get to school or work, and fit in social and physical activities before they return home in the evening. Most participants mentioned that they have to get up a little earlier to take their treatment and that when they are particularly busy they do sometimes forget.

In the morning I’m normally in a rush. So that’s when I normally tend to have it, so that’s why I tend to forget. I struggle to keep track of the days as well. (P.3, 21 years old)

Next to forgetting, one of the main barriers for the participants in this study is the intravenous injection itself, as it is not pleasant to have to inject regularly. And although participants did not describe any issues around needle phobia, it appears quite common for patients to experience some issues around venepuncture or venous access. Not being able to find or access a vein can be frustrating, painful and can sometimes cause anxiety. Some patients described how they tend to give up on their injection if it doesn’t work the first or second time, and come back to it later in the day or even the following day.

Sometimes when you put the needle in, sometimes it can be quite difficult. Cos you can’t always don’t always find the eh find the ehm the vein. Don’t always find it. (P.4, 12 years old)

Once they have found one or two injection sites that work well, most patients tend to stick to those sites for all their injections. This makes it easier and less painful, particularly as the sites become less sensitive due to the scar tissue that results from frequent injections.

I could obviously just go into another vein. But that would just be going into tender skin then. (P.3, 21 years old)

Sometimes there are practical reasons why patients struggle to take their treatment. For instance, they may not be able to inject themselves because of an injury to their arm. In emergencies patients may not always be able to mix up or take treatment themselves. The design of the treatment, and the way it is mixed up in preparation for the injection, can make it challenging for inexperienced people to help. This makes emergencies more stressful, and may increase general anxiety about managing life with haemophilia. One participant described an experience during a ‘lads holiday’, which has made him more anxious and risk averse about going away with friends or attending parties that may involve alcohol and young people having fun.

‘I’m going to have to go back to the room to treat myself, can one of you come and help me?’ my mates offered and we went back. It was hard for me to do it because it was bleeding, so I had to describe to my friend how to make the bottle up to get the injection ready. Obviously I don’t expect my friend to inject me. But I thought if you make it up for me I could put this extra pressure on and give myself a bit more time to make it stop. So when I come to inject and go like that then the blood will be minimal compared to if I just make it and the blood flow is going down. So I was telling him but it was slightly complicated. For me it’s second nature, but obviously for him I was just … trying to take it on board what I was giving to him. But if it was just maybe an easier way of making it or less compartments. Sort of readymade so all you have to do is inject then it would be like simpler. I could have just got on with it. (P.10, 21 years old)

Because prophylactic treatment involves an intravenous injection, it is important to find a private and clean place to do their treatment. This can be challenging when on holiday, or out and about.

I’ve never had factor on a plane before, and then we get in the airport and it’s hustle and bustle, you can’t really pull the lads to one side say “I’ve got to go to the toilet and do it”, so ‘I’m not really going to have treatment here until I get home’. So it was just the fact that I couldn’t have any, that was more the worry. It made me feel a bit not organised, not good about myself. (P.10, 21 years old)

*Subtheme 2.2: Haemophilia and treatment-related anxiety.* As described above, anxiety can make the intravenous injections more challenging, particularly for patients who have venous access or venepuncture issues.

What makes treatment difficult is if I’m in – If I’m stressed. If I had a stressful day and I’m doing it in the evening it’s difficult to locate veins. Because they tend to shy [laughs] away from you. If I’m in a bad mood they shy away. I – it’s always very mood specific. If I’m quite relaxed and n a good mood then I can easily see them on the surface and it’s easy to get in there. (P.1, 17 years old)

Although haemophilia teams work with patients from a young age to support and encourage them to take over responsibility for their injections gradually, younger participants who were not doing their own injections yet appeared a little anxious about the idea of taking over their own injections.

I have to one day obviously but it’s a bit scary, but I do have to eventually. (P.8, 12 years old)

The majority of participants explained that doing their own injections was difficult to start with, but became easier with time and experience. None of the participants appear to have developed needle phobia, although there was some indication that some of them may not have been completely at ease with their treatment. Several participants explained that they would never take their treatment in front of someone else, or would only do it in front of people they are familiar with.

Generally I’ll always do it before I go out or work is okay because you’re familiar with work so, I don’t like doing it in front of like, I don’t mind doing it in front of but, you know, people that don’t know about it and don’t know what it is, that’s, so generally I always make sure I have it in a safe place at home, have it at work, have it at a friend’s house … . (P.10, 21 years old)

Not wanting to take treatment in front of others indicates that there is some anxiety about how others respond to the injection, and possibly some concern about stigma. Some participants described how reactions from work colleagues and potential partners can be upsetting or cause insecurity.

Whereas some girls maybe I’ve seen in the past they’re like ‘what is it? What is it? You have to inject yourself’, you know, ‘I don’t understand why’ and makes you look a bit like, you know, it shows you up, makes you feel like, you know, I’m not good enough because I’ve got to inject myself or because it looks bad and as soon, normally generally the girls, I explained to them and said ‘look this is what it is, this is what I have to do’ and they’re fine. (P. 10, 21 years old)

It is clear that even for patients who have supportive families and friends around them, haemophilia can cause anxiety at times, particularly in cases where a bleeding episode causes them to miss school, work or social occasions. Although many patients described a certain level of pragmatism and not wanting to dwell on issues (see above), for some, particularly younger patients, haemophilia-related issues can become overwhelming at times.

Like if I have a bleed and I’m resting, I sometimes go on the Xbox and just like play and kind of forget about like the bleed. (P.8, 12 years old)

Some described how they would ‘flee’ from their haemophilia for a while by becoming absorbed in a film or computer game, others engaged in more active coping mechanisms like speaking to friends or seeking psychological support to help them deal with issues related to their haemophilia.

I do have counselling as well. And it all must stem back to when it all started or whenever. That’s just another problem I think. Just for someone to talk to, it is helpful. But I don’t like to – I don’t like to talk about a lot of things. I keep a lot to myself, so that’s why I go there. So yeah, haemophilia does cause a few problems in that way. Yes, it’s all about that, obviously growing up with it. (P.3, 21 years old)

Some participants were able to access psychological support through their haemophilia centre directly, or through referral to a psychological support service provided by the hospital or local NHS trust. However, long waiting lists or difficulty in accessing support meant that some participants sought a private counsellor or therapist. Regardless of how they accessed the support, participants felt it was very helpful to discuss their haemophilia-related issues with someone outside of their family, social circle or haemophilia team. Several participants made unprompted suggestions that it would be helpful if support was easier to access. They felt that in an ideal world psychological help would be available through haemophilia centres, so that patients could receive support without delay and without incurring costs. Support from family, peers, friends and the haemophilia team will be addressed in more detail below under theme 3.

*Subtheme 2.3: Non-adherence is usually due to forgetting.* Despite the treatment being quite unpleasant, self-reported adherence among participants in this study was high. Very few participants admitted to deliberately skipping treatments, and those participants who did would usually only skip a treatment if they were expecting to have a very inactive day. As described above, these circumstances are not necessarily considered as non-adherent for those participants who have agreed a more tailored treatment regimen with their consultant.

We do sometimes just not bother with the treatment, if I’m not going to be doing anything that day. (P.9, 16 years old)

None of the participants felt that adherence was an issue for them, and most were motivated to keep to their treatment regimen although some admitted to sometimes forgetting treatments, due to their busy lifestyle or being distracted by other priorities.

… over really important stuff it’s no, I don’t miss it. But I’ve had the odd kind of day where I’ve gone uhm. Where I’ve had an active day and I’ve got halfway through it and bugger it, I didn’t take my medication. So I’ll either go home and take it or if I just be super careful then … So I think you know maybe we’re talking twice a year I make a mistake with it. (P.2, 24 years old)

Yeah, I occasionally do forget it … if I have forgotten during the day mum would say have you done it? If not, right, you do it tomorrow morning then. (P.4, 12 years old)

Most participants take their treatment as soon as they realise they have forgotten it, others wait until the next morning. There were two participants who, despite reporting high adherence, described situations where they may miss two, three or even four injections in a row. It was not clear what the actual level of adherence of these participants was, and the language they used indicated that perhaps they were a little confused about this themselves.

No I – I won’t deliberately miss two three or four in a row, and if – if – the problem is if I forget one – the little dull aches a bit, yeah ok, if I miss two then they get worse, then it – then that’s it. Because you realise, yeah I’ve missed two, I need to get it done. And even if it’s a day when I’m not treating, I’ll treat myself for it. Because I can’t get these bruises. (P.1, 17 years old)

Some participants described being quite anxious or worried about the risk, when they realise they have forgotten their treatment. They clearly feel that their treatment protects them, and feel vulnerable when they realise they are not covered. For these participants, this anxiety could also act as an extra motivator to adhere, as it appeared that these participants forget very rarely.

… for me it’s a really horrible feeling to be doing something active having not had my medication, because then you know you’re vulnerable. (P.2, 24 years old)

One participant in particular described how forgetting treatment affects his mood, and makes him feel disappointed in himself. He feels happy when he has had his treatment, as he knows he is protected. But when he has forgotten his treatment he feels exposed and anxious.

A bit like exposed, feel a bit like, you know, a bit disappointed in myself in a way, sort of a bit like I should have had some before and I feel a bit like anxious to get it done really and I go in a bit of a bad mood or I go a bit, you know, feel a bit uncomfortable and not as happy as usually would be. (P.10, 21 years old)

*Subtheme 2.4: Taking treatment is inconvenient and no fun.* It is very clear that none of the participants enjoy taking their treatment, and would much prefer not to have to take it. Many participants mentioned that they do not like having to inject themselves intravenously, and would much prefer to take a tablet or subcutaneous injection.

Like instead of doing the injection you just gotta take a tablet. I know that you can’t do that but … . (P.4, 12 years old)

… like I wish it could be like a diabetic who gets it in a pen? (P.11, 24 years old)

One of the most frequently mentioned issues around the treatment was that it can be quite time-consuming. As most patients take their treatment in the morning it can be difficult to fit it in, and some participants admitted to sometimes having to miss breakfast when treatment takes a bit longer than usual, or when they do not have time for both treatment and breakfast.

The prophylaxis only really takes about 20 minutes but I don’t really have time for breakfast afterwards so it’s quite irritating before school but most of the time it’s not too bad. (P.9, 16 years old)

Interestingly, there was one participant who really disliked having to mix the treatment. He was not concerned about the injection itself, but felt mixing up treatment is boring and tedious and resented having to do it before every injection. He explained that his parents would often mix his treatment for him when he was growing up.

Especially when you’re growing up sometimes, like once a week for someone to mix it for you so you ain’t got to worry about it, it’s just ‘here you are, I’ve done it’, ain’t giving it, it’s just mixing it, especially when there was a lot of it, it was really tedious. (P.11, 24 years old)

*Subtheme 2.5: Taking treatment is part of my routine.* Although many participants admitted that they dislike their treatment, they also appeared to have accepted it as part of their life and have fitted it in their routine. For most, it has become just another thing they need to do before they leave home in the morning.

… it’s become second nature. It’s an easy thing to do now. I get up early in the morning and I sit – sat at the breakfast bar in there and I just sit and do it. I don’t even give it a second thought, you put it together and inject. (P.1, 17 years old)

It’s uhm it’s just something that’s gotta be done. It’s like it’s like having a shower in the morning. That’s pretty much the long and short of it. (P.2, 24 years old)

Participants described how the injections were often difficult and painful when they were younger, particularly when they were learning how to do the injection themselves. But by the time they reach young adulthood they tend to be very skilled, and able to inject themselves without any issues.

Yeah, I can do it with my eyes closed I reckon [both laugh] if I had to I think I could. (P.5, 25 years old)

In recent years factor replacement treatment has improved significantly, which has made it much easier for patients to keep to their regimen. Key improvements highlighted by participants in this study include the reduction in volume (making it easier to mix up and requiring smaller syringes); easier to store and transport (it does not require refrigeration and the boxes are smaller) and the improved instructions on the packaging (particularly helpful in emergencies when other people may need to help).

None of the participants suggested that these improvements have had a direct positive influence on their adherence, particularly as their adherence was generally already quite high. However, they felt that taking their treatment has become a lot easier and less time-consuming, which in turn may reduce negative associations with treatment and therefore reduce the risk of patients skipping treatments.

It’s a lot smaller now, so it’s a lot easier. You haven’t got to take these massive bottles which takes hours to mix up. It’s all very easy, you squirt in, you squirt out, and it’s all very quick these days (P.5, 25 years old).

It used to it had to be kept like fridge cold. But now it’s more just under more like room temperature which is fine. Which makes life quite easy (P.2, 24 years old).

As described above, non-adherence in this patient group tends to be due to forgetting. Many participants described easy and practical solutions that help them to remember their treatment, such as visual reminders (e.g. putting the bright yellow sharps bin on the breakfast bar); linking treatment to particular activities (such as having breakfast); and digital reminders (alerts on a mobile phone, tablet or computer).

It appeared that they were keen to find their own solutions and ways of managing their treatment, as everyone is different and has their own challenges to overcome. For instance, while some were keen to use their smartphones for reminders, another participant explained that he would never trust a digital device alone without a back-up reminder.

So it would make a noise or whatever, so you’d know it was that day. But that’s just then trusting a device to tell you when to do it without thinking … . (P.3, 21 years old)

Despite the various reminders, and the motivation to keep to their regimen, some participants admitted that they do occasionally forget their treatment. This is often because they live busy lives, and are sometimes distracted by other priorities. However, they tend to have some treatment stored at work or school so that they can take it there in case they have forgotten it at home. Having some treatment stored at work or school provides an extra safety net, which seemed particularly important for those participants who do not want to take any risks.

… but I’ve also got some at work. You know, just in case I need it. Like in an emergency, which I’ve had to before. Or just in case I forgot to take some with me, so there’s always some there. (P.3, 21 years old)

*Subtheme 2.6: Treatment protects me so I can live a normal life.* Many participants explained that they feel their prophylaxis protects them, and allows them to live a normal life. For some that means that they feel protected in everyday activities, such as going to school or work. Others feel that prophylaxis supports them in living an active lifestyle, including competing in top-level sports.

*It’s helping me lead a normal life. (P.2, 24 years old)*

The treatment works quite well, it stops most bleeds. It’s only the serious ones that get through now. We do a big dose before and after the activity … I’ll still get a small bleed but nothing like as bad as I would get if we didn’t. (P.9, 16 years old)

Participants explained that they top-up to cover themselves for sport and physical activities. Most seemed to recognise that in addition to reducing the risk of bleeds, prophylaxis helps to prevent long-term issues such as joint damage.

If I don’t take my medication I’ll start getting internal bleeds that’s going to affect me later in life with joint damage and what not. (P.2, 24 years old)

#### Theme 3: Support from family, friends and the haemophilia centre keeps me on track

Self-management for haemophilia includes a number of behaviours, some of which can be quite challenging. The majority of participants felt that they would not be able to look after themselves without support from their parents and the staff at the haemophilia centre. This theme covers all the forms of support that were described by participants.

*Subtheme 3.1: Support from mum and dad is key.* Parental support is particularly important in relation to adherence. As described above, in the majority of families it is the parents who are responsible for their child’s haemophilia treatment for the first 12 years or so. This means they have to inject their child several times each week, log each treatment, arrange for treatment to be delivered, arrange regular hospital check-up appointments, and be on stand-by just in case their child experiences a bleed at school.

I think they was always worried and mum would come into school every now and then to give me treatment and things if I needed, if I fell over in the playground or, you know, when I was younger they’d come in and they’d say, you know ‘I’m here, we need to do this, we need to do that’. (P.10, 21 years old)

Parental support appears particularly important in encouraging adherence, as parents tend to remind their child to take his treatment every time it is due. Some parents continue to remind their child even after they has left home; this appears to be a habit of a lifetime that is difficult to break. Many parents also continue to do the actual injection every now and then, just to share the burden and make life a little easier. Parents often also stay involved with arranging the deliveries of treatment, and continue to help when bleeds or other health issues occur.

Now that I am a bit older I am quite self-sufficient with it, but uhm cos I travel a fair bit so my mum looks after, the treatment gets delivered to my mums house rather than mine, cos mum is in a lot more often than I am. She’ll keep an eye on my stock I’ve got. Uhm so, yeah they are still. Yeah everyone supports me really nicely. (P.2, 24 years old)

*Subtheme 3.2: Social and peer support.* In addition to support from parents and siblings, participants described supportive social networks they could rely on for practical and emotional support.

And especially with the friends who I’ve been on holiday with they understand it a lot better because I understand their problems, they understand my problem, we know what our limitations are – and we can just stick together. (P.1, 17 years old)

However, as described above, some participants felt that they had missed out on opportunities to develop social skills due to their haemophilia. Some reported that they therefore found it difficult to develop supportive relationships and struggled to ask for or receive social support. These participants generally continued to obtain their support from within the family (specifically parents).

Advice about what activities children with haemophilia should avoid has changed in recent years, and many children are now allowed to take part in physical education lessons at school as long as they avoid contact sports such as rugby and hockey (Farrugia, Gringeri, & von Mackensen, 2018; Howell, Scott, & Patel, 2017). Any activities that may cause head injury need to be avoided at all costs, as intracranial bleeding represents one of the biggest risks. However, it is unlikely that young children who are growing up with haemophilia today are excluded from playing outside at break time at school. Hopefully, this means that this new generation is able to take part in most activities at school, and does not miss socially. This in turn may help them develop social skills and form supportive friendships. This certainly appeared to be the case for the younger participants in this study, who described very supportive friendships.

It doesn’t really bother me that much now because most of my friends at school help me. It feels all right because most of my friends know about it and they to talk to me about it. (P.8, 12 years old)

Sometimes when I’m at school I do talk about it or like if I say I’m in pain they normally help me and take me to a teacher. (P.4, 12 years old)

One form of social support that may be particularly helpful is peer support from other young people with haemophilia (Omura et al., 2013). As it is so rare, patients do not often meet other people who have haemophilia (Khair & Holland, 2013). One participant joked that he always felt that he was the only person with haemophilia. Now that he is in his twenties he receives psychological support from a counsellor to talk about his haemophilia and the way it has affected him. Some haemophilia centres have peer support groups, and organise social outings to give young people with haemophilia (YPH) the opportunity to meet and socialise. Pressure on budgets make it hard to keep these activities going, and in some centres they have now stopped. Participants indicated that they had benefitted from opportunities to interact with other YPH outside the hospital setting.

*Subtheme 3.3: The staff at the haemophilia centre encourage me to keep to my treatment regimen.* The majority of patients appear to have a good relationship and regular contact with their haemophilia centres. Sometimes this is just informal contact to check how patients are doing, and other times it may be to provide support for specific issues. It appears that HP in the centres involved in this research study are able to recognise when particular individuals need help or additional support.

Yeah, it’s nice to know, they ring up and ask and they’re concerned and, you can, obviously it was a mistake on my behalf so I said like no, and they told me how to do it properly and generally it was, the nurses are good I think, yeah, they’re okay. They help out as much as they can and it was good that they’re keeping an eye on me really. (P.10, 21 years old)

Nurses appear to keep a close eye on patients, particularly those who suffer from regular bleeds. They check Haemtrack (a secure online treatment log that enables patients to record all therapies as they occur, and allows clinicians to see up-to-date information to help monitor, optimise and improve patient care) to monitor individual patients. In many cases where a review of Haemtrack reveals that a patient may not be adhering to their treatment regimen, or has had a serious bleed without contacting the centre, nurses contact the patient to discuss potential issues. This appears to encourage patients to adhere to their prophylaxis, and stay on top of managing any bleeds.

Generally the nurse will ring and say ‘you’ve had a lot of factor, we’re looking on your Haemtrack, you’ve had a lot’, I think one time they’d mistaken, I had this ankle injury but I kept putting it was a follow-up bleed, because it was the same sort of bleed reoccurring again, but in fact I should have done new bleeds every times, so it looked like I’d had this bleed for like nine hundred hours or something. So then they rang up and was like ‘look, is this bleed this bad or is it because it’s reoccurring but is it because it’s a new bleed?’. (P.10, 21 years old)

It appears that many haemophilia centres use the carrot and stick approach, where they combine regular encouragement and support with a telling off when patients are not adhering to their treatment.

I know definitely you do get the same motivation when every time I go for the clinic appointments, trust me, I do get a big lecture! (P.6, 22 years old)

Regardless of how the different haemophilia centres approach different patients, all participants in this study were very positive about the healthcare and support they receive. It is clear that many nurses, haematologists, psychologists, physiotherapists and other allied health professionals often go out of their way to support their patients.

Support from the centre makes me feel less alone. (P.3, 21 years old)

The nice thing for me is I know they’re there if I need them. (P.2, 24 years old)

Support from family, friends and haemophilia team helps young people to keep on track with their treatment. Parents appear to be very involved with their sons’ haemophilia management, even after they leave home. Help and support from the haemophilia team appears an important facilitator to adherence.

## Discussion

The present study examined adherence to prophylaxis in interviews with 11 young people with haemophilia, recruited from 5 haemophilia centres across England and Wales.

The findings suggest that many young patients experience a tension between their desire to be ‘normal’ and the successful self-management of their haemophilia. Self-reported adherence to prophylaxis was good, with few participants admitting to intentionally skipping injections although forgetting was sometimes reported. However, the findings also indicate that due to the increasingly personalised and flexible approach to prophylaxis, adherence is not always straightforward to define. Several participants appeared slightly confused when describing their own levels of adherence, particularly when they were asked to distinguish between forgetting and skipping.

The main barriers to adherence are the time and effort needed to take treatment (fitting this into an already busy lifestyle); dislike of the intravenous injection; venous access issues; anxiety or stress (haemophilia-related as well as general); and being out of the normal routine (e.g. on holiday)(Schrijvers et al., 2013; van Os et al., 2017).

Support from family, friends and haemophilia team helps young people to keep on track with their treatment. Parents appear to be very involved with their sons’ haemophilia management, even after they leave home. Help and support from the haemophilia team appears an important facilitator to adherence.

### Strengths and limitations

Data represent the lived experience of individual participants. Existing quantitative measures do not permit the study of adherence to prophylaxis in an increasingly personalised treatment approach. It was crucial, therefore, to examine this issue using a qualitative approach in order to determine how adherence is experienced in relation to the everyday lives of patients, fitting around roles, identities, lifestyles and relationships. The use of IPA to analyse interviews was especially important since it does not rely on measuring previously formed attitudes of which participants are consciously aware but, instead, involves the researcher making sense of participants making sense of their experiences.

One potential limitation is that the participants were not necessarily representative of the whole population of young people with haemophilia. It is possible that those who agreed to be interviewed are more motivated and engaged in relation to their treatment. Those who are non-adherent or disengaged are less likely to take part in research about adherence. Nevertheless, participants were recruited from five haemophilia centres across England and Wales, including paediatric, adult, and mixed centres. These centres are very different, not only in terms of their geographic location, but also their organisational structures. This allowed the researcher to gain insights into experiences across a range of treatment contexts.

The focus in this study is specifically on young people rather than including patients of all age groups. It is important to focus on one age-group because there are age-specific issues that may influence adherence, such as historical changes in types of treatment available (e.g. most young people with haemophilia alive today have followed a prophylactic regimen from childhood compared to older patients who did not have this treatment available when they were young), and factors associated with different developmental and life stages. Research using a different age-group may therefore identify different issues influencing adherence.

A final limitation is that the majority of interviews took place within hospitals. Although the interviews were conducted by a psychologist who was not involved in participants’ haemophilia treatment, and in a private room, patients’ responses may have been influenced by their previous (potentially stressful or traumatic) experiences in the hospital.

### Implications

Notwithstanding the limitations discussed above, the findings of this study have a number of implications. The results indicate that adherence among young people is generally good, particularly in comparison to adherence among young people diagnosed with chronic health conditions (DiMatteo, 2004; KyngÄs, Kroll, & Duffy, 2000), such as diabetes (Iannotti et al., 2006; Griva et al., 2000; La Greca et al., 1995), juvenile arthritis (Salema et al., 2011), and asthma (McQuaid, Kopel, Klein, & Fritz, 2003). Support from parents and the haemophilia team appear to be the most important facilitators to their adherence. Haemophilia teams appear to work hard to maintain a good relationship with patients, particularly with those patients who are struggling. In the current economic climate, and in the context of reorganisation and rationalisation of the NHS, this model of care may come under increased pressure and may not be able continue if resources are cut. However, the findings from this study provide evidence for the benefits of the current approach in managing patients with severe haemophilia, and may therefore help haemophilia centres to build a case to retain their current level of resource.

Many patients require, and indeed access, additional support from a psychologist to help them with the psychological and social impact of haemophilia. This psychological support appears to be an important element of the comprehensive care that many patients require (‘Feeling angry? Frustrated? Anxious? Low?,’)*.* Participants felt that this support should ideally be provided within the haemophilia centre, so that the threshold to access this support is low and psychologists are able to work with patients pro-actively to address issues before they escalate. However, several of the centres involved in this research no longer have (or never had) a psychologist embedded in their team. The findings of this research may help centres to put forward an argument to improve access to psychological support, or indeed appoint a psychologist in their centre.

The findings also indicate that, due to the increasingly flexible approach to haemophilia treatment in the UK, adherence to prophylaxis is difficult to define and assess. Patients’ tendency to bleed is not only determined by the severity of their haemophilia and extent to which they adhere to their treatment, but also by their bleeding phenotype, which can be relatively mild even for patients with severe haemophilia. Therefore it could be that a patient with severe haemophilia ‘gets away’ with non-adherence to a certain extent, and may not necessarily need to improve their adherence. The findings of this research suggest that it may be useful to shift the focus of future research away from looking for ways to improve adherence generally, but rather to focus on improving adherence among those patients who are likely to have worse outcomes due to sub-optimal adherence.

In a quantitative study (van Os et al., 2017) non-adherence was more likely to be due to forgetting than skipping. Although it is likely that young people’s busy lifestyles are partially responsible for this, findings from the present study indicate that patients may find it easier to admit to forgetting than skipping because they feel they are more likely to be ‘told off’ if they admit to intentionally skipping treatments.

The increasingly flexible approach to prophylaxis, which gives some patients the freedom to change the timing of their injections, is likely to make it more complicated to keep track of when injections are due. Rather than following a strict routine, in which injections are done on the same day each week, or every other day, patients with a flexible regimen will need to manage their treatment actively, remembering when the last treatment was done and when the next one is due. Not having a well-established routine may lead to unintentional non-adherence in some cases. A more flexible approach to prophylaxis can also make it more challenging for the haemophilia team to ascertain the extent to which individual patients are adhering to their regimen. Going forward, dichotomising non-adherent patients into ‘forgetters’ and ‘skippers’ may not very useful when designing interventions aimed at improving adherence. As regimens are often tailored, it follows that approaches aimed at improving adherence may also need to be tailored around individual circumstances. It appears that in many circumstances this is already the case, as haemophilia teams tend to work with individual patients in a way that suits their personal situation.

Another important development to consider in relation to adherence to prophylaxis is the recent introduction of extended half-life factor products, which can significantly reduce the number of intravenous infusions needed, whilst also in many patients aim for increased protection from debilitating bleeding events (need to add reference). Switching to a regimen with less frequent injections is likely to facilitate the use of prophylaxis in patients with poor venous access and may perhaps increase adherence among some patients due to the reduced treatment burden. However, it may reduce adherence in other patients, for instance those who are more likely to forget if they do not follow a strict routine.

The personalised approach to prophylaxis raises some interesting questions in relation to adherence. In some instances behaviour that would have been considered non-adherent just a few years ago (e.g. reducing number of injections during a ‘quiet’ week), may now be seen as an acceptable adjustment. Equally, the definition of ‘over-treating’ may also need to be revised, as patients who increase the dose or frequency of their injections to accommodate a more active lifestyle are not necessarily over-treating. This has some important implications for patient education and information, adherence assessment and research, and haemophilia care. Patient education about treatment is likely to become more complicated, as tailored treatment regimens are more difficult to explain and require patients to have a better understanding of how the treatment works. Assessing adherence will also become more complicated, as standard adherence questionnaires used in research may not accommodate a more flexible treatment, and assessment of adherence in clinical settings will be more complicated and time-consuming.

## Conclusion

The current approach that haemophilia teams follow to support patients in managing their haemophilia treatment is working well. The increasingly flexible and personalised approach allows patients to tailor their treatment around their lifestyle and personal circumstances. This in turn motivates them to keep on track with their treatment, which in turn results in reduced bleeding episodes and its associated costs for patients, the NHS, and the wider society. Personalised prophylaxis dosing, both in terms of timing and pharmacokinetic-guided dosing is increasingly relevant in haemophilia centres. However, this flexible approach can also lead to some confusion around treatment frequency, dosing and may lead to accidental non-adherence. Some additional training and education of patients and their families to increase their knowledge and skills around prophylaxis may help to improve adherence, and thus protection, among those patients who currently miss occasional treatments due to misunderstanding personalised-tailoring around their activities.
